# Are Adolescents Sensitive About Sensitive Data? Exploring Student Concerns About Privacy, Confidentiality, and Data Use in Health Research

**DOI:** 10.1016/j.jadohealth.2025.03.004

**Published:** 2025-05-15

**Authors:** Emma Soneson, Mina Fazel, Puneetha S. Goli, Simon R. White

**Affiliations:** a Department of Psychiatry, University of Oxford, Oxford, United Kingdom; b UT Southwestern Medical School, Dallas, Texas; c Department of Psychiatry, University of Cambridge, Cambridge, United Kingdom; d MRC Biostatistics Unit, University of Cambridge, Cambridge, United Kingdom

**Keywords:** Adolescents, Mental health, Methodology, Research methods, Study design, Survey, Intervention

## Abstract

**Purpose:**

We quantitively explored adolescents’ concerns about privacy, confidentiality, and data use in health research and their potential impact on the accuracy of self-report data.

**Methods:**

We analyzed data from 17,729 secondary school students who participated in the 2023 OxWell Student Survey. The survey assessed 5 concerns about privacy, confidentiality, and data use and asked students whether these concerns impacted the accuracy of their answers. We calculated the proportions who (a) endorsed each concern and (b) reported inaccuracies associated with their concern(s). We then examined associations of concerns and self-reported inaccuracies with nonresponse and score distributions on sensitive measures of mental illness (depression/anxiety and disordered eating) and adversity (child maltreatment) using logistic regression.

**Results:**

46.0% (8,160/17,729) of students endorsed ≥1 concern, and of these, 29.2% (2,379/8,160) reported associated inaccuracies. Relative to boys, concerns were more common amongst gender diverse adolescents (adjusted odds ratio [aOR] = 5.71, 95% confidence interval [CI] 4.40–7.48), gender nondisclosing adolescents (aOR = 4.36, 95% CI 3.62–5.26), and girls (aOR = 2.52, 95% CI 2.36–2.69), with smaller differences in self-reported inaccuracies. Students with self-reported inaccuracies were significantly more likely to have nonresponse on the 3 measures of mental illness and adversity (aORs = 1.53–3.38), whilst score distributions on those measures varied substantially according to whether students reported concerns.

**Discussion:**

Concerns about privacy, confidentiality, and data use were common amongst student participants, as were self-reported inaccuracies. Substantial differences in nonresponse and score distributions on sensitive measures highlight potential impacts of these concerns. Co-designing and implementing strategies to address these concerns might help to support evidence-based decision-making by improving representativeness and data quality in adolescent health research.

Empowering adolescents to take part in research and provide accurate self-reported responses about their health and well-being is critical for understanding their needs and informing the design and implementation of tailored interventions, services, and policies. However, despite the importance of hearing directly from adolescents on matters relating to their own lives, some adolescents choose not to participate in research, and many are hesitant about what they disclose if they do take part [1].

Adolescents face several potential barriers when deciding whether or how to participate in research [1–6], many of which may be shaped by their (dis)trust of researchers or the research process [6,7]. For many, privacy, confidentiality, and uncertainty about how their data will be used are foremost concerns [3,5–8], particularly in terms of how data are stored [9] and who might see their responses either when taking part (e.g., a peer seeing their answers whilst completing a school-based survey) or afterward (e.g., the research team sharing their answers with a parent or teacher) [3,5,7]. For some adolescents, these concerns might discourage them from participating in research altogether. For others who *do* ultimately decide to participate, such concerns may influence the way they respond by, for example, providing inaccurate responses in the context of self-report measures [10] or declining to respond to particular questions altogether [11].

Understanding these concerns and their potential impact is especially relevant for research that may be perceived as sensitive. Defining ‘sensitive’ research is challenging, as there is considerable variation in the way adolescents understand and express their thoughts, feelings, and experiences, and what is ‘sensitive’ for one adolescent may not be ‘sensitive’ for another. However, many areas of particular interest and relevance to policy and practice [1,3] – such as mental health, self-harm, suicidality, violence and abuse, sexual activity, and illegal or risk-taking behaviors – have been highlighted as potentially sensitive areas [1,3,8,10,12]. Adolescents’ concerns may be especially relevant in these contexts and could, in theory, lead to systematic nonparticipation if adolescents are worried about reporting on sensitive topics. There is evidence to suggest this may be the case, as previous studies have found that adolescents involved in certain risk behaviors (e.g., smoking or alcohol or substance use [13]), those with antisocial behaviors (e.g., bullying [14]), and those with poorer health and well-being [13,15] are often under-represented in research.

In addition to nonparticipation, research that asks participants to report on sensitive topics may also have a heightened risk for inaccurate data, whereby some participants may decline to answer or provide inaccurate responses, such as ones that they perceive will not trigger criminal or safeguarding action or that are more ‘socially desirable’ [10,16]. A broad body of literature across health and social sciences has explored the potential for inaccuracies on sensitive items in questionnaire-based research using approaches such as comparing responses collected using anonymous versus confidential procedures [17], examining the impact of experimentally-manipulated levels of trust and privacy on responses [18], assessing variation according to self-reported survey items about accuracy/honesty [16,19,20], validating self-reported behaviors against biological measures [21], and comparing survey responses with administrative data [22]. However, in general, these studies have not been designed in a way that enables them to empirically explore the potential reasons for any inaccuracies discovered.

In understanding how concerns about privacy, confidentiality, and data use may impact adolescent health research, it is also important to consider how they are distributed throughout the population, as this may have implications for developing strategies to encourage engagement. Notably, there is a paucity of direct evidence (i.e., from asking adolescents themselves) as to whether some adolescents are more likely than others to have such concerns, but indirect evidence extrapolated from differential consent and attrition suggests that concerns might be unequally distributed across sociodemographic groups. For example, previous studies have demonstrated higher rates of nonparticipation amongst children and adolescents from lower socioeconomic backgrounds; those with minoritized ethnic, sexual, and gender identities; and those with mental health difficulties, particularly where active parental consent was required [4,15,23–25]. There are several potential reasons why some groups of adolescents may be less likely than others to engage with health research when invited, including lack of trust in the research process, perceptions of limited benefit for their respective communities, or concerns about how they will be perceived by their peers and/or the research team [1].

Adolescents’ concerns can adversely impact the quality of research and any conclusions drawn from data collected. Concerns that influence adolescents’ decisions of whether to participate or how to engage with research can reduce both sample size and precision of effect estimates. Furthermore, systematic variations in the prevalence or impact of concerns across subgroups of the population, if present, are cause for particular concern, as they may contribute to selection and/or attrition biases that decrease sample representativeness, with downstream implications for how evidence is used to inform practice.

## The current study

High quality research that captures accurate information about adolescents’ lives, experiences, and preferences is paramount for better understanding and supporting their health and well-being. Accurate data are particularly important for informing policy and practice, as decisions based on poor quality data can have suboptimal – and even adverse – outcomes. Yet, with a few notable exceptions [3,5], there has been limited systematic exploration of adolescents’ experiences of and concerns about participating in health research. A better understanding of adolescents’ perspectives could help us not only to identify potential biases in self-report data, but also to design and implement strategies that facilitate recruitment, retention, and accurate data capture. To further develop this evidence base, we used data from a large school-based survey to address the following 4 research questions:

RQ 1. How common are adolescent concerns about privacy, confidentiality, and data use in research and self-reported inaccuracies due to these concerns?

RQ 2. Are there differences in concerns and self-reported inaccuracies by gender, key (educational) stage, and ethnicity?

RQ 3. Are concerns and self-reported inaccuracies associated with *nonresponse* on sensitive measures of mental illness and adversity?

RQ 4. Are concerns and self-reported inaccuracies associated with *scores* on sensitive measures of mental illness and adversity?

## Methods

### Data source: The OxWell Student Survey

This study used data from the secondary school version of the 2023 OxWell Student Survey [26], a self-report repeated cross-sectional survey examining the health and well-being of students in English schools and further education colleges. Schools were recruited directly by local authorities and enrolled students using (1) a parental opt-out model and student assent (student age <16 years) or (2) students’ informed consent (student age ≥16 years) only. The survey was administered during the school day and did not collect identifiable information to increase the diversity and representativeness of the sample, facilitate a parental opt-out model to ensure maximum participation, and encourage more accurate and honest responses [26]. The University of Oxford Research Ethics Committee granted ethical approval for the study (Reference: R62366/RE014).

### Measures

The full OxWell variable guide is available at osf.io/bwech and questions from our sensitive measures of mental illness and adversity are also presented in Table S1.

#### Concerns and self-reported inaccuracies

On the final page of the (relatively long) survey, students were asked to share about their experience of participation with options to endorse as many potential concerns as were relevant to their experience. This analysis focused on concerns pertaining to privacy, confidentiality, and data use (henceforth ‘concerns’), namely: ‘I was worried that an adult at school/college would see my answers’ – ‘I was worried that friends or peers would see my answers’ – ‘I was worried that someone at home would see my answers’ – ‘I did not believe that the survey was actually confidential’ – ‘I was worried about how my answers would be used (e.g., in research)’. If a student endorsed any of these options, they were then asked *for each endorsed concern* ‘Did this prevent you from answering 100% accurately?’ (‘yes’ – ‘no’ – ‘prefer not to say’). These questions have iteratively evolved since 2019, when we drew on existing literature, consultation with a Young People’s Advisory Group (YPAG), and our team’s wider research interests to derive a series of questions to explore the potential value of making the survey more identifiable (see study protocol [26]).

#### Sensitive measures

We selected 3 of OxWell’s validated measures to serve as exemplar outcomes from a longer list of topics our YPAG felt might be particularly sensitive (see *Youth involvement*). These assessed depression and anxiety, disordered eating, and child maltreatment. *Depression and anxiety:* the 11-item Revised Children’s Anxiety and Depression Scale (RCADS-11) [27] is comprised of depression (5 items) and anxiety (6 items) subscales (‘Never’ [0] to ‘Always’ [3]) and 2 additional items assessing impact (‘Not at all’ [0] to ‘A great deal’ [3]) for a total score range of 0–39, with higher scores representing greater symptoms/impact. *Disordered eating:* the Development and Well-Being Assessment (DAWBA) [28] contains 5 screening questions pertaining to eating disorders, with binary ‘yes’ (1) and ‘no’ (0) response options, for a total score range of 0–5, with higher scores representing greater disordered eating. *Child maltreatment:* the Short Child Maltreatment Questionnaire (SCMQ) [29] measures experience of physical, emotional, and sexual abuse; physical and emotional neglect; and exposure to domestic violence. OxWell uses 6 of the scale’s 7 items (excluding one of the 2 sexual abuse items). For each item, response options are ‘No, never’, ‘Yes, it has happened in my life’, ‘Yes, it has happened in the last 12 months’, and ‘Prefer not to answer’. For any response of ‘yes’, students are asked whether the experience has occurred ‘Once or twice’ or ‘Many times’. We generated a sum score by coding an experience as 0 if the student had never experienced it, 1 if they had experienced it ‘once or twice’ (in the last year and/or in their lifetime), and 2 if they had experienced it ‘many times’ (in the last year and/or in their lifetime). Total scores therefore ranged 0–12, with higher scores indicating more types and/or greater frequency of maltreatment.

#### Sociodemographic characteristics

Sociodemographic characteristics included gender, key (educational) stage, and ethnicity. We processed gender responses into categories of ‘girl’, ‘boy’, ‘gender diverse’, ‘gender nondisclosing’ (i.e., those who chose ‘prefer not to say’), and ‘unsure’, and treated free-text answers deemed ‘likely disingenuous/not gender’ as missing data. The detail of this processing is described in a separate publication [30], but in brief, it involved a process of iterative coproduction with 3 trans and gender diverse young people whereby we (1) systematically excluded free-text responses that were likely indicators of students not taking the survey (or at least the gender question) seriously or who misinterpreted the question (indicated, for example, by answers pertaining to sexuality or religion) and (2) derived meaningful and inclusive categories based on the remaining responses. Key stage (KS) refers to the way English school years (equivalent to ‘grades’ elsewhere) are grouped. KS3 groups Years 7, 8, and 9 (representing a typical age range of 11–14 years), KS4 groups Years 10 and 11 (representing a typical age range of 14–16 years), and KS5 groups Years 12 and 13 (representing a typical age range of 16–18 years). Ethnicity was collected using 18 response categories and was collapsed into 5 groups (White; Asian/Asian British; Black/Black British/African/Caribbean; Mixed/multiple ethnic groups; and other ethnic group) according to 2021 Census Classification 6a [31].

### Analysis

*RQ 1*. We calculated the proportion of students endorsing concerns and associated self-reported inaccuracies. *RQ 2*. We explored demographic differences in gender, KS, and ethnicity using logistic regression. For this analysis, we dichotomized the total number of concerns and self-reported inaccuracies into ‘none’ or ‘any’ (i.e., ≥1). For the main analysis, we considered answers of ‘yes’ as self-reported inaccuracies, but additionally conducted a series of sensitivity analyses (for RQs 2 and 3) wherein self-reported inaccuracies are also reported for those who responded ‘yes’ *OR* ‘prefer not to say’, based on an assumption that answers of ‘prefer not to say’ may be more likely to represent inaccuracies than answers of ‘no’. *RQ 3*. We used logistic regression to determine whether concerns and self-reported inaccuracies were associated with nonresponse across each of our 3 sensitive measures. For the purposes of this analysis, we were interested in *any* missingness, so rather than assessing nonresponse as the number (count) or proportion of missing items, or as missingness on selected items hypothesized to be ‘most sensitive’, we defined our nonresponse outcome as missingness of ≥1 item for each of the 3 questionnaires. *RQ 4*. We explored distributional shifts in scores on the 3 sensitive measures according to whether students reported concerns and associated inaccuracies and used the Anderson-Darling test to determine whether each group of students was drawn from the same distribution. By definition, students with any missing data on the 3 measures were excluded from this analysis. All analyses were conducted in R (version 4.1.2) and the code is available at is available at https://github.com/simon-r-white/are_adolescents_sensitive_about_sensitive_data.

### Youth involvement

As part of this analysis, we held 2 online sessions with a YPAG comprised of 14 (Session 1) and 12 (Session 2) adolescents aged 16–18 years. In the first session, we invited the attendees to complete a ranking activity that asked them to identify which topics in the OxWell survey they considered to be most ‘sensitive’ and a sorting activity about how likely young people were to respond inaccurately to each topic. The discussions in this first session, alongside statistical considerations, helped inform our selection of the 3 sensitive measures used in the present analysis. In the second session, we presented the findings from our analysis in an accessible and interactive way, asked about their interpretation, and discussed implications for adolescent health research. This included facilitated discussions to brainstorm ideas for addressing adolescents’ concerns as well as a ranking exercise based on a hypothetical scenario about response accuracy. The discussions in this session helped contextualize our findings and suggest potential avenues to empower adolescents to more meaningfully participate in research.

## Results

### Sample characteristics

The 2023 OxWell Student Survey collected 34,245 responses from secondary school and further education college students (approximate ages 11–18 years). We excluded 4,974 students who did not assent/consent or minimally engage [26] and 11,542 students who likely did not reach the end of the survey, where they were asked about their concerns (the survey required 30 minutes on average to complete, which was not possible in all settings). We therefore consider a subsample of 17,729 students from 77 secondary schools and further education colleges (Table 1). In terms of gender split, 52.4% of students identified as girls, 42.0% as boys, and 1.6% as gender diverse, and a further 3.1% were gender nondisclosing (i.e., chose ‘prefer not to say’). The majority of students (58.7%) were in KS3, 27.6% were in KS4, and 13.7% were in KS5. The sample was ethnically diverse: just over half (54.4%) of students identified as White; 14.9% as Asian or Asian British; 5.6% as mixed or multiple ethnic groups; 4.3% as Black, Black British, Caribbean or African; and 3.6% as belonging to another ethnic group. Nonresponse was high, with 17.2% choosing not to disclose their ethnicity.

### Findings

#### RQ 1. How common are adolescent concerns about privacy, confidentiality, and data use in research and self-reported inaccuracies due to these concerns?

**Figure 1:**
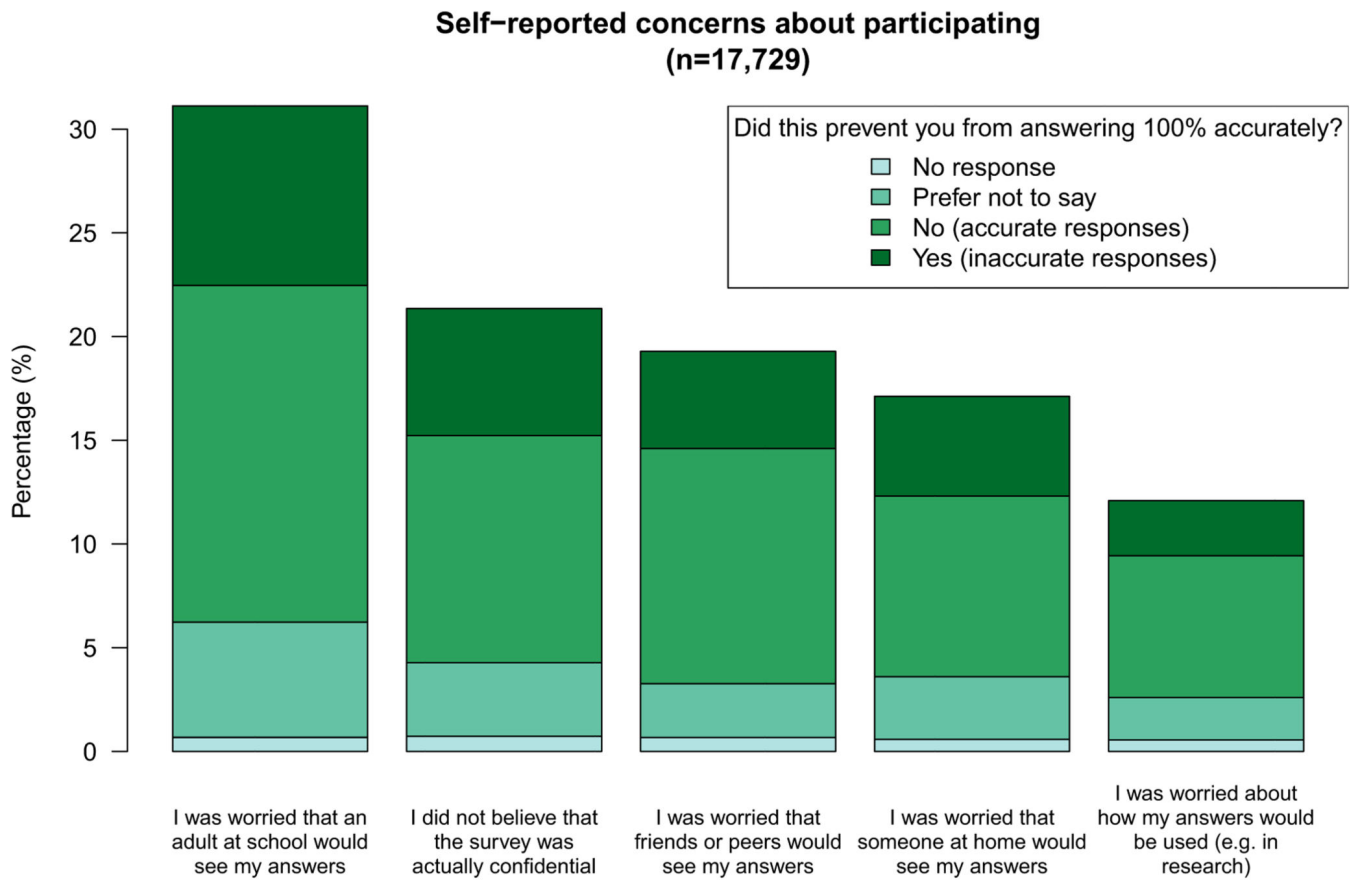
Proportions of students reporting concerns about privacy, confidentiality, and data use, and self-reported inaccuracy due to the concern(s).

In total, 46.0% (8,160/17,729) endorsed at least one of the 5 concerns, and 28.8% (5,300/17,729) endorsed at least 2 (Figure 1, Table S2). Students’ most common concern was that an adult at school would see their answers (31.1%; 5,518/17,729), followed by a concern that the survey was not actually confidential (21.4%; 3,786/17,729). In the subsample of those with at least one concern, when asked whether their concern(s) impacted the accuracy of their answers, 29.2% (2,379/8,160) responded ‘yes’ at least once (in our sensitivity analyses, where we considered answers of ‘yes’ *OR* ‘prefer not to say’, this figure increased to 46.2% [3,770/8,160]). The proportion self-reporting inaccuracies was relatively consistent across concerns, ranging from 21.9% (concern about how their answers would be used, 470/2,143) to 28.7% (concern that the survey was not actually confidential, 1,086/3,786; Table S2).

#### RQ 2. Are there differences in concerns and self-reported inaccuracies by gender, KS, and ethnicity?

Compared with boys, gender diverse adolescents (adjusted odds ratio [aOR] = 5.71, 95% confidence interval [CI] 4.40–7.48), gender nondisclosing adolescents (aOR = 4.36, 95% CI 3.62–5.26), girls (aOR = 2.52, 95% CI 2.36–2.69), and students who provided no response to the gender question (aOR = 2.05, 95% CI 1.13–3.72) were more likely to endorse ≥1 concern (Table 2). Amongst those with ≥1 concern, gender diverse adolescents were less likely than boys (aOR = 0.67, 95% CI 0.47–0.95) to report inaccuracies due to their concern(s). However, there were notable differences in gender patterns in the sensitivity analysis (Table S3). In the sensitivity analysis, relative to boys, self-reported inaccuracies were more common amongst gender nondisclosing adolescents (aOR = 1.53, 95% CI 1.23–1.91) and girls (aOR = 1.31, 95% CI 1.19–1.45), whereas there was no significant difference for gender diverse adolescents.

Whilst there were statistically significant differences across KSs, there was no clear pattern in terms of direction. For ethnicity, Asian or Asian British students and those from ‘Other’ ethnic groups were each slightly less likely than White students to have any concerns (aOR = 0.90, 95% CI 0.82–0.99 and aOR = 0.81, 95% CI 0.68–0.96, respectively). Black, Black British, Caribbean or African students (aOR = 1.33, 95% CI 1.06–1.67) and those who did not disclose their ethnicity (aOR = 1.23, 95% CI 1.08–1.40) were more likely than White students to report inaccuracies due to their concern(s). In the sensitivity analysis, only those who did not disclose their ethnicity were more likely than White students to report inaccuracies (Table S3).

#### RQ 3. Are concerns and self-reported inaccuracies associated with nonresponse on sensitive measures of mental illness and adversity?

**Figure 2:**
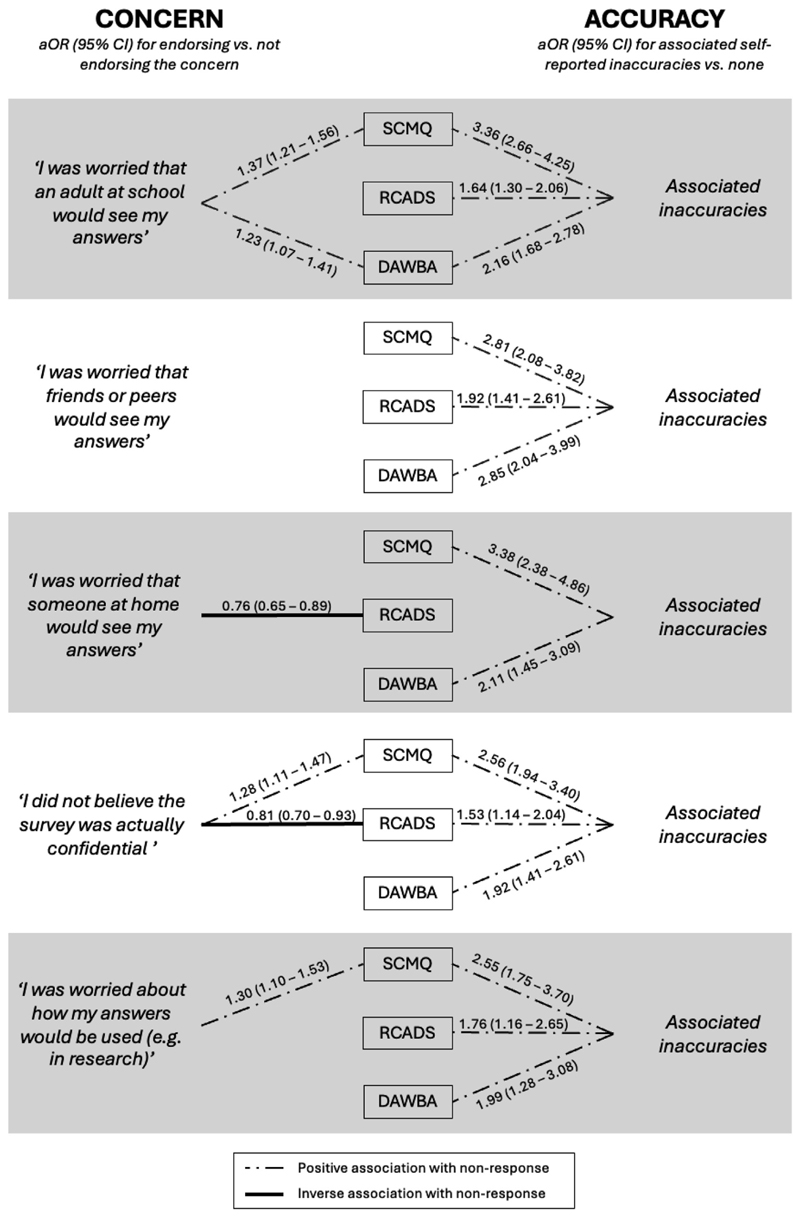
Summary of associations between each of the 5 concerns (left) and self-reported inaccuracies due to the concerns (right) and nonresponse on 3 sensitive measures. Dashed lines indicate that those who endorsed the concern/inaccuracy were more likely to have nonresponse, and solid lines indicate that they were less likely to have nonresponse. All associations were adjusted for gender, key stage, and ethnicity. Full results are available in Table S4. DAWBA = Development and Well-Being Assessment eating disorder screening questions (5 total items); RCADS-11 = 11-Item Revised Children’s Anxiety and Depression Scale (13 total items); SCMQ = Short Child Maltreatment Questionnaire (6–12 total items, depending on answers).

Figure 2 overviews the associations between concerns, self-reported inaccuracies, and nonresponse on the 3 sensitive measures of mental illness and adversity (adjusted for gender, KS, and ethnicity); Table S4 provides full results.

Associations between the presence of concerns and nonresponse on the RCADS-11, DAWBA eating disorder screening questions, and SCMQ were inconsistent and in most cases nonsignificant: of a possible 15 associations (5 concerns *x* 3 sensitive measures), 9 were nonsignificant. In terms of significant associations, students who were concerned that an adult at school would see their answers were *more* likely to have nonresponse on the SCMQ (aOR = 1.37, 95% CI 1.21–1.56) and the DAWBA questions (aOR = 1.23, 95% CI 1.07–1.41); those concerned about how their answers would be used were *more* likely to have nonresponse on the SCMQ (aOR = 1.30, 95% CI 1.10–1.53); those who believed the survey was not confidential were *more* likely to have nonresponse on the SCMQ (aOR = 1.28, 95% CI 1.11–1.47) but *less* likely to have nonresponse on the RCADS-11 (aOR = 0.81, 95% CI 0.70–0.93); and those concerned about someone at home seeing their answers were *less* likely to have nonresponse on the RCADS-11 (aOR = 0.76, 95% CI 0.65–0.89).

In contrast, there was a clear and consistent relationship between self-reported inaccuracies and nonresponse, whereby adolescents with self-reported inaccuracies were, with one nonsignificant exception, more likely than their peers with ≥1 concern but no self-reported inaccuracies to have nonresponse on the 3 sensitive measures (aOR = 1.53–3.38). This relationship was especially apparent for the SCMQ and DAWBA eating disorder screening questions. The strongest association was between self-reported inaccuracies due to a concern about someone at home seeing the student’s answers and nonresponse on the SCMQ (aOR = 3.38, 95% CI 2.38–4.86). For this research question, patterns in the sensitivity analysis were nearly identical those in the main analysis (Table S4).

#### RQ 4. Are concerns and self-reported inaccuracies associated with scores on sensitive measures of mental illness and adversity?

**Figure 3:**
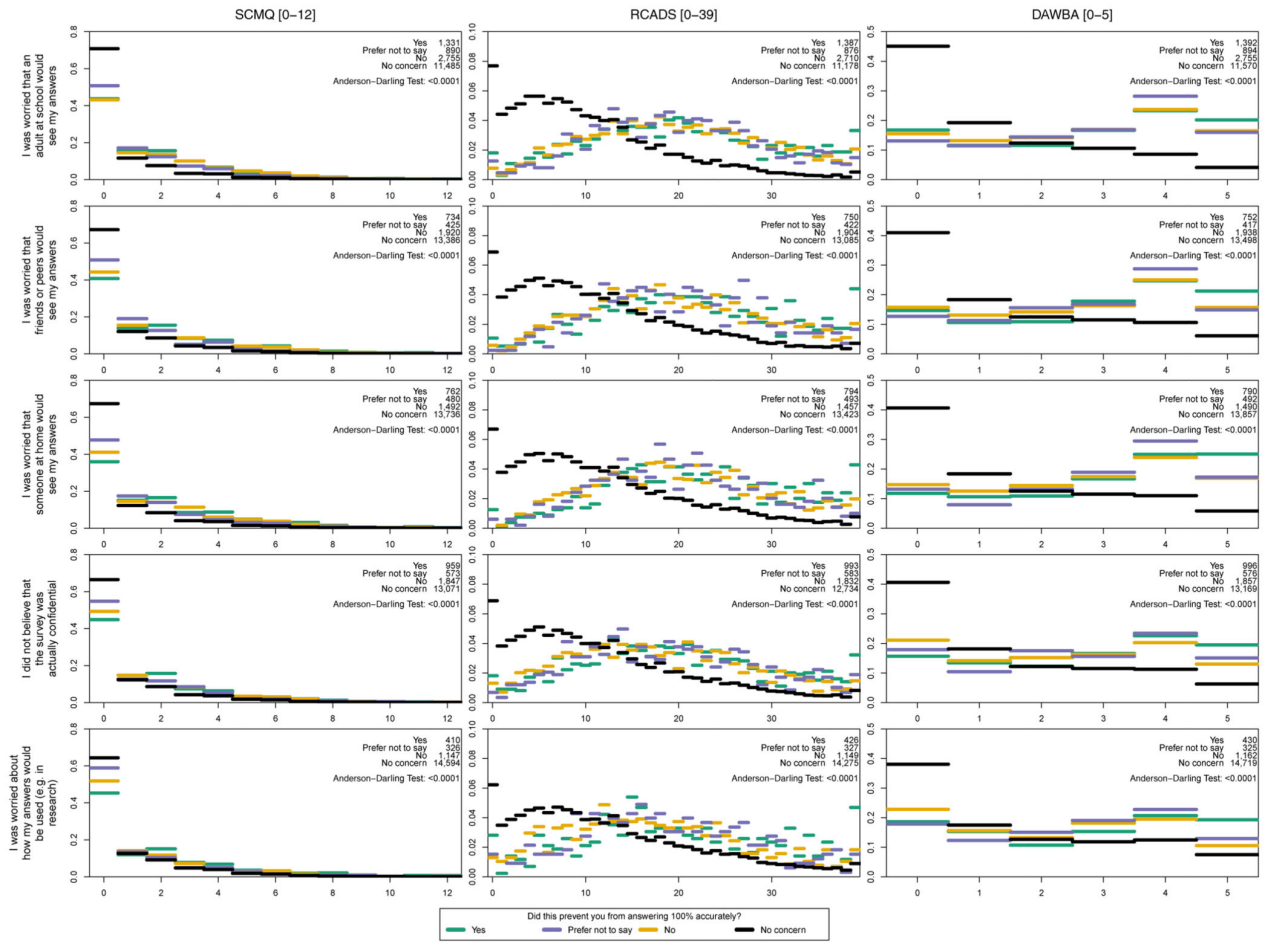
Score distributions for 3 potentially sensitive measures. DAWBA = Development and Well-Being Assessment eating disorder screening questions (scored 0–5); RCADS-11 = 11-Item Revised Children’s Anxiety and Depression Scale (scored 0–39); SCMQ = Short Child Maltreatment Questionnaire (scored 0–12).

Score distributions on the SCMQ, RCADS-11, and DAWBA eating disorder screening questions showed notable differences between students who endorsed each concern and those who did not (Figure 3). Across all 5 concerns, the scores of students *with* the concern were concentrated at the moresevere end of each measure (i.e., more maltreatment, depression and anxiety, and disordered eating) compared with students *without* the concern. Amongst those endorsing each concern, there was relatively less difference according to whether or not they reported inaccuracies.

As an example, the RCADS-11 score distributions suggest that students who were concerned that an adult at school would see their answers (teal, purple, and yellow lines) had substantially higher depression and anxiety scores than their peers without that concern (black line). However, amongst the students with that concern, there was less difference in score distributions according to whether students self-reported inaccuracies in their answers (i.e., the teal, purple, and yellow distributions were largely similar). Importantly, it is not possible to draw conclusions about causality (e.g., that having this concern *led or contributed to* higher depression and anxiety scores, or that having higher depression and anxiety scores *led or contributed to* the presence of the concern).

## Discussion

Concerns about privacy, confidentiality, and data use (‘concerns’) were common in a large, nonidentifiable, school-based survey of adolescent health and well-being. Nearly half of students (46.0%) endorsed at least one of the 5 concerns examined, and nearly a third of these (29.2%) reported that their concern(s) had influenced the accuracy of their answers. There were prominent differences according to gender, whereby girls, gender diverse adolescents, and gender nondisclosing adolescents had between 2.52 and 5.71 times the odds of boys of endorsing at least one concern. Interestingly, differences in self-reported inaccuracies were only evident amongst gender diverse adolescents, who were less likely than boys to report inaccuracies. In terms of our 3 sensitive measures, which assessed depression and anxiety, disordered eating, and child maltreatment, there was no clear relationship between the presence of concerns and nonresponse, whereas those who self-reported inaccuracies were substantially more likely to have missing data on each of the measures (aORs = 1.53–3.38). Score distributions on the 3 sensitive measures varied substantially according to whether students endorsed a concern, but not by self-reported inaccuracies. Those with concerns demonstrated higher levels of vulnerability (i.e., greater mental illness and adversity) than their peers.

The high proportion of adolescents reporting concerns about taking part in research underscores the importance of privacy and confidentiality in this group [3,5–8]. Critically, our estimates are likely to under-represent the prevalence of concerns in the young population. Our questions on concerns were located at the end of the survey, meaning that we captured information *only for students who reached the last page*. We were therefore not able to measure concerns for those who stopped partway through (either by choice or due to time constraints), or perhaps more importantly, those who did not assent/consent to participate at the outset, who arguably may be the most likely to have research-related concerns. Although many studies have assessed sociodemographic differences in (non)participation in research [13,15,25], few have systematically and directly assessed adolescent concerns about privacy, confidentiality, and data use [9], even though doing so can provide insight into specific reasons why adolescents might decline opportunities to participate. For both groups – those who choose not to participate and those who participate but have reservations – qualitative research may help better understand the nature of their concerns and how best to address them.

Our findings suggest that, in order to leverage the strengths of self-report methods for capturing information about adolescents’ internal and external worlds [32], those designing research with adolescents need to be deliberate about addressing their concerns, especially when examining more sensitive and delicate issues. Our YPAG had several ideas for doing so. For example, they suggested that meeting the research team directly to hear about their motivations might foster trust, which could encourage more meaningful participation and which has been shown empirically to predict better engagement with sensitive questions [18]. To help with concerns about data use and confidentiality, they suggested providing students with an illustrated data ‘pipeline’ showing how research data are used and shared and who is involved at each stage. One potentially important element of the pipeline is being clear about what happens with sensitive data specifically (e.g., whether and in which circumstances safeguarding action could be taken), as our YPAG highlighted that some adolescents might fear that their answers could trigger criminal or safe-guarding action. Finally, they suggested logistical solutions to concerns about school staff or peers seeing answers during survey administration, such as ensuring privacy and security in the physical spaces where any research takes place. Ongoing integration of – alongside dedicated funding for – the youth voice in research may support participation and enhance data integrity, not only for individual projects but for the field more widely.

The findings also shed light on how concerns might impact data integrity. As described in the wider literature [10,11,16] and echoed by our YPAG, adolescents who have concerns might take several different approaches to the survey, including not answering items or providing answers that were more socially desirable. Interestingly, we found contrasting patterns for nonresponse and score distributions on the 3 sensitive measures: variations in score distributions were most pronounced according to the *presence/absence of concerns*, whereas variations in nonresponse were most pronounced according to *self-reported inaccuracies* (although, again, it is important to note that those with missing data were excluded from the score distribution analysis). This suggests that having a concern and reporting associated inaccuracies may impact data quality in diverse ways, building on findings from studies demonstrating differential response patterns on measures of suicidal thoughts, depression, and risk behaviors according to student responses on self-reported honesty items [19,20,33]. However, whilst others have suggested that these adolescents are not taking the research ‘seriously’, we propose that such patterns may also arise due to concerns about how their participation might be monitored by their school, how their data will be used in the context of the research, and what potential repercussions there might be if they disclose information that they perceive could require a formal (e.g., safeguarding) response.

Estimates based on inaccurate data may have adverse consequences for evidence-based decision-making in adolescent health and well-being [1,20], and our findings have important implications for data integrity. First, we need to have heightened awareness that adolescents completing questionnaires might not answer specific questions or might answer inaccurately if they do not understand or trust how their data will be used and/or shared. At a population level, inaccurate data, such as under-reporting of psychopathology or child maltreatment, can lead to misallocation of limited resources. This is especially worrying where concerns and their impacts are unequally distributed amongst the adolescent population, as seen, for example, in the gender differences identified in this study, as this may lead to biases in the findings which, when used to inform policy and practice decisions, could exacerbate pre-existing inequalities. Second, whilst not directly examined in this analysis, it is also possible that at an individual level, adolescents’ concerns about how their data might be used (not only within services but also within their families and their schools) might lead to potential over-reporting or under-reporting. For example, if an adolescent does not want involvement from services, they might under-report symptoms, their severity, or their impact, whereas the opposite may be true for an adolescent who *does* want additional support but is worried they might not reach clinical thresholds.

### Strengths and limitations

We used a large, diverse sample to explore the prevalence and potential impacts of concerns about taking part in health and well-being research in an understudied age group. Our dual focus on (1) endorsing a concern and (2) reporting that the concern influenced accuracy allowed us to begin to tease apart the nuanced ways in which concerns might influence participation and engagement. Of note, OxWell’s parental opt-out model provided us with enhanced insight into adolescents’ own views, as studies with parental opt-in consent often exclude adolescents who might otherwise be willing to participate [25]. Finally, our consultations with the YPAG allowed us to explore which areas of health and well-being adolescents might find most sensitive, how they might interpret the question about answering ‘accurately’, and what might be done in the future to encourage research participation.

In terms of limitations, we were not able to capture concerns for those who participated but did not finish the survey, those who chose not to answer that question, or those who did not participate at all (a potential reflection of heightened concern). There are also limitations associated with our question on inaccuracies. Although we intentionally chose to frame it around ‘inaccuracy’ to avoid the negative connotations of ‘dishonesty’, some students might have had unintended interpretations of that term, which may have influenced our results. The question also did not specify *which* questions students answered inaccurately nor *the way* in which they answered inaccurately (e.g., left blank, chose more socially desirable answers, answered randomly, etc.) Furthermore, the fact that the survey asked a separate question about accuracy for each endorsed concern presented analysis and interpretation challenges. First, in our regression models, adolescents who endorsed a higher number of concerns would have had more opportunities to report inaccuracies (e.g., a student who endorsed all 5 concerns would have had 5 opportunities to report inaccuracies whereas a student who endorsed one concern would have had only one opportunity); we believe our decision to dichotomize inaccuracy outcomes to ‘none’ versus ‘any’ to be the most appropriate and interpretable approach. Second, we did not ask an overall question about accuracy, so it is possible that some students who did not endorse any of the 5 concerns may still have answered inaccurately on some of the questions, which our data would not have identified.

Another limitation pertains to high missingness on our ethnicity variable (17.2%). This missingness might be masking associations with ethnicity, for example if students who worried about identification due to being one of a small number of students of a given ethnicity in their school were less likely to disclose their ethnicity. Finally, for RQ 4, there was evidence of zero-inflated score distributions (i.e., a high proportion of students with scores of 0; Figure 3), which was difficult to interpret and which may benefit from further systematic investigation.

## Conclusions

High-quality, accurate research is essential for supporting evidence-based decision-making for adolescent health and well-being, yet concerns about research participation are notably common in this age group and may have implications for the quality of data collected and conclusions drawn. Researchers can potentially improve data accuracy and completeness by better understanding and addressing adolescents’ hesitations and concerns, ideally by working in partnership with adolescent advisors to encourage more meaningful and accurate participation, thus supporting better service development and design.

## Supplementary Material

Supplementary Data

## Figures and Tables

**Table 1:** Sample characteristics

Characteristic	N (%)
Gender	
Girl	9,296 (52.4)
Boy	7,450 (42.0)
Gender nondisclosing	549 (3.1)
Gender diverse	288 (1.6)
Unsure	9 (0.1)
Likely disingenuous/not gender	93 (0.5)
No response	44 (0.2)
Key stage	
KS3 (ages 11–14)	10,401 (58.7)
KS4 (ages 14–16)	4,896 (27.6)
KS5 (ages 16–18)	2,432 (13.7)
Ethnicity	
Asian/Asian British	2,645 (14.9)
Black/Black British/African/Caribbean	761 (4.3)
Mixed/multiple ethnic groups	984 (5.6)
Other ethnic group	635 (3.6)
White	9,648 (54.4)
No response	3,056 (17.2)

**Table 2:** Logistic regression models for (a) ≥1 concern about privacy, confidentiality, and data use and (b) any self-reported inaccuracy due to the concern(s)

Predictors	≥1 concern					Any self-reported inaccuracy (for those with ≥1 concern)			
	Unadjusted OR [95% CI]	*p*	Adjusted OR [95% CI]	*p*		Unadjusted OR [95% CI]	*p*	Adjusted OR [95% CI]	*p*
(Intercept)	–	–	0.48 [0.45, 0.51]	**<.001**		–	–	0.38 [0.34, 0.42]	**<.001**
Gender*									
Boy	Ref	–	Ref.	–		Ref	–	Ref	–
Girl	2.51 [2.36, 2.68]	**<.001**	2.52 [2.36, 2.69]	**<.001**		1.05 [0.95, 1.17]	.339	1.06 [0.95, 1.18]	.306
Gender diverse	5.62 [4.33, 7.36]	**<.001**	5.71 [4.40, 7.48]	**<.001**		0.64 [0.45, 0.90]	**.013**	0.67 [0.47, 0.95]	**.026**
Gender nondisclosing	4.39 [3.65, 5.29]	**<.001**	4.36 [3.62, 5.26]	**<.001**		0.93 [0.73, 1.19]	.565	0.92 [0.72, 1.17]	.506
Unsure [censored, N<10]	–	–	–	–		–	–	–	–
No response	2.09 [1.15, 3.79]	**.015**	2.05 [1.13, 3.72]	**.018**		1.43 [0.57, 3.35]	.425	1.37 [0.54, 3.23]	.480
Key stage (KS)*									
KS 3 (ages 11–14)	Ref	–	Ref.	–		Ref	–	Ref	–
KS 4 (ages 14–16)	1.15 [1.07, 1.23]	**<.001**	1.13 [1.05, 1.21]	**.001**		1.12 [1.01, 1.25]	**.031**	1.12 [1.01, 1.25]	**.038**
KS 5 (ages 16–18)	0.86 [0.79, 0.94]	**.001**	0.84 [0.77, 0.92]	**<.001**		0.75 [0.64, 0.88]	**<.001**	0.76 [0.65, 0.89]	**.001**
Ethnic group									
White (aggregated)	Ref.	–	Ref.	–		Ref.	–	Ref	–
Asian/Asian British (aggregated)	0.96 [0.88, 1.05]	.349	0.90 [0.82, 0.99]	**.023**		0.99 [0.86, 1.14]	.901	0.97 [0.84, 1.12]	.680
Black/Black British/African/Caribbean (aggregated)	1.03 [0.89, 1.20]	.684	0.99 [0.85, 1.15]	.870		1.36 [1.08, 1.70]	**.008**	1.33 [1.06, 1.67]	**.014**
Mixed/multiple ethnic groups (aggregated)	1.20 [1.06, 1.37]	**.006**	1.13 [0.99, 1.30]	.072		0.97 [0.79, 1.19]	.799	0.97 [0.79, 1.19]	.763
Other ethnic group	0.80 [0.68, 0.94]	**.007**	0.81 [0.68, 0.96]	**.014**		1.14 [0.87, 1.50]	.330	1.12 [0.85, 1.46]	.422
No response	0.96 [0.88, 1.04]	.278	1.01 [0.93, 1.10]	.731		1.23 [1.08, 1.41]	**.001**	1.23 [1.08, 1.40]	**.002**
Observations			17,729					8,160	
R^2^ Tjur			0.061					0.007	

Bold values indicate statistically significant at *p* < .05. CI = confidence interval; OR = odds ratio.

* ‘Key stage’ refers to groupings of English school years; the overlap in ages is due to the fact that a year group covers multiple ages according to what time of the yeara student was born.
