# Supplementary material for: Are Adolescents Sensitive About Sensitive Data? Exploring Student Concerns About Privacy, Confidentiality, and Data Use in Health Research
Source: J Adolesc Health. Author manuscript; Available in PMC 2026 Apr 22. (PMC7619027; doi:10.1016/j.jadohealth.2025.03.004)
Supplement: Supplementary Data [file EMS213317-supplement-Supplementary_Data.pdf]

Supplementary Data

Supplementary data related to this article can be found at https://doi.org/10.1016/j.jadohealth.2025.03.004.
